# Canine microfilaraemia in some regions of Iran

**DOI:** 10.1186/s13071-022-05209-7

**Published:** 2022-03-18

**Authors:** Seyed Hossein Hosseini, Fateme Manshori-Ghaishghorshagh, Mohammad Ramezani, Hassan Nayebzadeh, Mohammad Bagher Ahoo, Ahdieh Eslamian, Minoo Soltani, Shahram Jamshidi, Marcos Antonio Bezerra-Santos, Fatemeh Jalousian, Alireza Sazmand, Domenico Otranto

**Affiliations:** 1 Department of Parasitology, Faculty of Veterinary Medicine, University of Tehran, Tehran, Iran; 2 The Iranian Museum of Parasitology, Faculty of Veterinary Medicine, University of Tehran, Tehran, Iran; 3 Department of Pathobiology, Faculty of Veterinary Medicine, Lorestan University, Khorramabad, Iran; 4 Faculty of Veterinary Medicine, Rastegar Reference Laboratory, University of Tehran, Tehran, Iran; 5 Department of Internal Medicine, Faculty of Veterinary Medicine, University of Tehran, Tehran, Iran; 6 Department of Veterinary Medicine, University of Bari, Bari, Italy; 7 Department of Pathobiology, Faculty of Veterinary Science, Bu-Ali Sina University, Hamedan, Iran; 8 Zoonotic Diseases Research Center, School of Public Health, Shahid Sadoughi University of Medical Sciences, Yazd, Iran

**Keywords:** *Acanthocheilonema reconditum*, *Dirofilaria immitis*, Haemoparasites, Iran, PCR, Zoonosis

## Abstract

**Background:**

*Dirofilaria immitis* and *Dirofilaria repens* are vector-borne zoonotic parasites which affect mainly dogs and humans worldwide. In Iran, information about the distribution of those nematodes is scant in several regions. Therefore, we investigated the prevalence of these filarial parasites in stray dogs from five Iranian provinces where no information about these parasites is available.

**Methods:**

Blood samples were collected from 344 stray dogs in five provinces of Iran (i.e. Mazandaran, Gilan, Esfahan, Qazvin and Loresan). The presence of microfilariae was assessed using direct smear, modified Knott’s test, molecular detection of filarial DNA (*cox*1 gene) and *Wolbachia* endosymbiont of parasitic nematodes (*ftsZ* gene) by conventional PCR (cPCR). All of the PCR products were sequenced and phylogenetic analysis was performed.

**Results:**

In total, 75 dogs (21.8%) were found to be positive for *D. immitis* by cPCR. Infection was detected in all provinces, with the highest prevalence in Gilan province (22/28; 78.6%). *Acanthocheilonema reconditum* was diagnosed in five dogs (1.4%) from three provinces (i.e. Esfahan, Mazandaran, Gilan). Two dogs were infected with both parasites and three were only infected with *A. reconditum*. *Dirofilaria repens* infection was not found in the examined population. Representative sequences of the *D. immitis cox*1 gene from dogs from the northern provinces (Mazandaran, Gilan, Qazvin) were grouped together and distinctly separate from the ones from western and central provinces (Lorestan and Esfahan), suggesting that different nematode populations are present in the country.

**Conclusion:**

The data reported herein fill existing gaps in knowledge about canine filarial infection in two Iranian provinces and record the highest prevalence of *D. immitis* ever reported in the country (i.e. 78.6%). A geographical review of the literature about *Dirofilaria* spp. and *A. reconditum* infections in dogs and humans has also been summarized, indicating that *D. immitis* and *D. repens* are distributed in 22 of 31 provinces in Iran, whereas *A. reconditum* is present in fewer regions. Effective control strategies are advocated for owned dogs, and a national program for the management of stray dogs is needed to minimize the risk of infection in animals and humans.

**Graphical Abstract:**

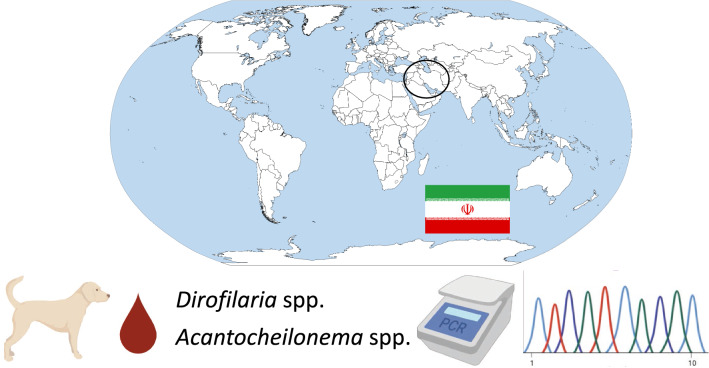

## Background

Canine dirofilariosis infections caused by *Dirofilaria* spp. (Spirurida, Onchocercidae) are a group of worldwide-distributed vector-borne diseases (VBDs) transmitted by over 70 species of mosquitoes of the genera *Aedes*, *Ochlerotatus*, *Anopheles* and *Culex* [1]. *Dirofilaria immitis* and *Dirofilaria repens* are the best known agents of canine dirofilariosis and may also occasionally infect other animal species, including cats and humans [2]. While *D. immitis* infection, also known as heartworm disease (HWD), may cause severe conditions (e.g. respiratory distress, epistaxis, haemoptysis, ascites and exercise intolerance), *D. repens* is less pathogenic, being localized under the skin of the infested animals [3]. Analogously, *Acanthocheilonema reconditum*, which is transmitted by fleas (e.g. *Ctenocephalides canis*, *Ctenocephalides felis*) and lice (e.g. *Heterodoxus spiniger*, *Linognathus setosus*), is a less pathogenic filarial species [4], with adult worms residing in the peritoneal cavity [5]. Although *A. reconditum* is not considered a major zoonotic risk, in a single case report, it was removed from the subconjunctival space in a patient from Australia [6].

Since the first report of canine dirofilariosis in Iran [7], *D. immitis* and *D. repens* have been recorded in dogs, wolves, jackals, foxes and cats in 18 of the 31 provinces [8–10], along with at least 22 human cases including subcutaneous, ocular and pulmonary presentations, and a rare case of testicular hydrocele [9, 11]. In Iran, *Culex theileri* has been identified as a vector of *D. immitis* by dissection of mosquitoes collected in Meshkinshahr county in the northwest [12]. In addition, DNA of *D. immitis* has been identified in *Simulium turgaicum* sensu lato [13]. It has been estimated that 11.5% of dogs in Iran are infected by *D. immitis*, which is slightly higher than the estimated global prevalence of 10.9% [14]. However, an infection rate of 62.8% was reported from dogs of Meshkinshahr [15]. Several human cases of *D. repens* infection have been reported in Iran; however, data for dogs are scant [7], and few instances of circulating microfilariae have been documented [16, 17].

Since its first record in six dogs [18], *A. reconditum* microfilariae have been reported from dogs in many regions in Iran [19–21]. However, due to its morphological similarity to *D. immitis*, it could be misidentified, leading to a lack of information about the real picture of the distribution and prevalence of this nematode in the country. Overall, despite some reports on the prevalence of canine *Dirofilaria* spp. and *A. reconditum* in Iran [9, 21], comprehensive epidemiological data would help in implementing control measures and reducing zoonotic risk. In this study we investigated the prevalence of this filarial species in stray dogs from five Iranian provinces where no information about these parasites is available.

## Methods

### Study area, sampling and laboratory procedures

**Fig. 1:**
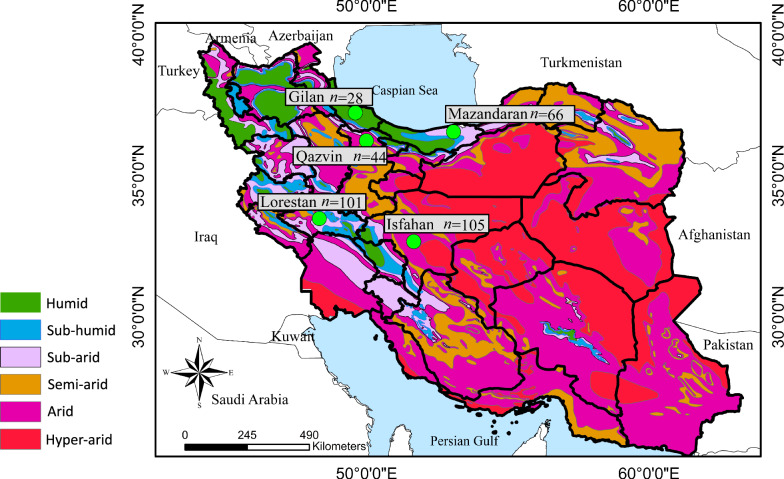
Samples were collected from five provinces in Iran with different climates

From December 2016 to December 2018, blood samples (2 ml) were collected from the cephalic or saphenous veins of stray dogs (*n* = 344; non-probabilistically sampled by convenience [22]) from five provinces of Iran including Mazandaran, Gilan and Qazvin in the north, Esfahan in the centre and Lorestan in the west, which are areas with different climatic characteristics (Fig. 1). Animal data (i.e. age, sex and province) were also recorded and were grouped according to age as ≤ 1 year (G1), > 1 to < 5 years (G2), and ≥ 5 years (G3).

Urban dogs were captured and transferred to the shelters of either the Diseases Control Office affiliated to the Provincial Veterinary Organization or the Provincial Department of Environment, where the data for animals were recorded. Based on the microfilaraemia periodicity for *D. immitis* and *D. repens* [23, 24], blood collections were performed from 11 p.m. to 1 a.m. On the following day, dogs were moved to the registered shelters in each province.

Blood samples were tested for circulating microfilariae using light microscopy observation of Giemsa-stained thin blood smears and the concentration method by modified Knott’s test. Microscopic examinations were performed under a BA410 Elite Trinocular light microscope (Motic Ltd., Xiamen, China) equipped with a digital camera (IDS uEye UI-2250SE USB 2.0 camera, Obersulm, Germany). The number of microfilariae per microlitre was calculated as described in a previous study [25]. Measurements of microfilariae were performed for up to 10 microfilariae of each nematode species per infected dog using AxioVision^*®*^ software version 4.1 (Zeiss, Göttingen, Germany).

### Molecular procedures

Genomic DNA was extracted from aliquots of 500 μl ethylenediaminetetraacetic acid (EDTA)-treated blood samples using a commercial kit (MBST, Tehran, Iran) following the manufacturer’s instructions. The DNA yield was assessed using a Thermo Scientific™ NanoDrop™ spectrophotometer (Thermo Fisher Scientific, Waltham, MA, USA). All samples were tested for the presence of *Dirofilaria* spp. and *A. reconditum* cytochrome *c* oxidase subunit 1 (*cox*1) using conventional polymerase chain reaction (cPCR) [25, 26]. Furthermore, *Wolbachia* DNA was detected by cPCR using primers targeting genes encoding filamenting temperature-sensitive mutant Z (*ftsZ*) [27] (Table 1). For all reactions, DNA of pathogen-positive blood samples served as a positive control and samples with no template DNA as a negative control. Amplified PCR products were visualized in 2% agarose gel stained with RedSafe™ (iNtRON Biotechnology, Gyeonggi, South Korea) using the microDOC Compact Gel Documentation System with UV Transilluminator (UVT312, Merck KGaA, Darmstadt, Germany). The PCR products were purified using CinnaPure DNA (CinnaGen, Tehran, Iran) and submitted for sequencing in both directions using an ABI 3730xl DNA Analyzer (Thermo Fisher Scientific, Waltham, MA, USA) at the Bioneer company (Daejeon, Republic of Korea). Nucleotide sequences were edited, aligned and analysed using the Chromas platform version 3.1 (Technelysium PTY Ltd., Queensland, Australia) and compared with those available in the GenBank^®^ database using the Basic Local Alignment Search Tool (http://blast.ncbi.nlm.nih.gov/Blast.cgi) and ClustalW (http://www.clustalw.genome.jp).

**Table 1:** Primers, target genes and PCR conditions used in this study

Pathogen	Primers	Annealing temperature (°C)	Target gene	Product size (bp)	References
*Acanthocheilonema reconditum*	AR COI-F1: AGTGTTGAGGGACAGCCAGAATTG AR COI-R1: CCAAAACTGGAACAGACAAAACAAGC	59	*cox*1	200	[25]
*Dirofilaria* spp.	COXdirHRMF: AGTATGTTTGTTTGAACTTC COXdirHRMR: AACGATCCTTATCAGTCAA	52	*cox*1	256	[26]
*Wolbachia pipientis*	Wol1_fwd: CCTGTACTATATCCAAGAATTACTG Wol1_R: ACTATCCTTTATATGTTCCATAATTTC	57.5	*ftsZ*	267	[27]

### Phylogenetic analysis

The *cox*1 and *ftsZ* representative sequences obtained in this study and the corresponding sequences available from the GenBank database were imported into MEGA X [28], and phylogenetic relationships were inferred using the maximum likelihood (ML) method and Tamura–Nei model [29]. Initial trees for the heuristic search were obtained automatically by applying neighbour-joining and BIONJ algorithms to a matrix of pairwise distances estimated using the maximum composite likelihood (MCL) approach, and then selecting the topology with the superior log-likelihood value. The tree is drawn to scale, with branch lengths measured in the number of substitutions per site. Codon positions included were 1st + 2nd + 3rd + Non-coding. Homologous sequences from *Wolbachia* endosymbiont of *D. immitis* (accession no. FJ390296) were used as outgroups.

### Statistical analyses

Exact binomial 95% confidence intervals (CIs) were established for proportions. Cohen’s kappa (κ) was calculated to compare the agreement between Knott’s modified test and PCR results. The Chi-square test and logistic regression analysis were used to compare proportions, with a probability *P*-value < 0.05 regarded as statistically significant. Analyses were performed using SPSS version 18 software for Windows 10 (SPSS Inc., Chicago, IL, USA).

## Results

Of the 78 dogs with a positive cPCR result for *D. immitis* (*n* = 75; 21.8%, 95% CI: 17.5–26.5) and for *A*. *reconditum* (*n* = 5; 1.45%, 95% CI: 0.47–3.36), two dogs were co-infected with both parasites and three were only infected with *A*. *reconditum*. Twenty-one dogs (6.1%; 95% CI: 1.88–1.95) were positive according to the modified Knott’s method and none based on Giemsa-stained blood smears. Based on the length and morphology of the microfilariae, infection with *D. immitis* or *A. reconditum* or co-infection with both species was diagnosed in 19, one and two dogs, respectively (Table 2). The number of microfilariae ranged from 0.05 to 1.85 mf/μl (SD ± 0.55). The length of microfilariae of *D. immitis* was 311.8 ± 9.8 μm (280–328) and for *A. reconditum* was 228.2 ± 12.1 μm (212–252) (Table 2). The level of agreement between the modified Knott’s test and cPCR was low for both *D. immitis* (*κ* = 0.35) and *A. reconditum* (*κ* = 0.57).

**Table 2:** Comparison of results from different diagnostic tools employed for the detection of blood microfilariae in 344 dogs in Iran

	*Dirofilaria immitis*	*Acanthocheilonema reconditum*
Direct blood smear	0	0
Modified Knott’s method	21 (8.8%)	3 (1.2%)
DNA of nematode	75 (23.2%)	5 (1.4%)
DNA of *Wolbachia*	75 (23.2%)	Not performed

The infection with *D. immitis* was present in all five regions, but *A. reconditum*-infected dogs were from three provinces, i.e. Mazandaran, Gilan and Esfahan. The risk of infection by *D. immitis* was significantly associated with the locality (Chi-square test, χ^2^ = 77.7, *df* = 4, *P* = 0.0001); that is, dogs in northern provinces (Gilan, Mazandaran and Qazvin) with temperate and Mediterranean climate had a higher chance of the presence of microfilaraemiae than those from the warm and dry provinces of Esfahan and Lorestan. Furthermore, age was statistically significant for *D. immitis* (Chi-square test, χ^2^ = 14.6, *df* = 2, *P* = 0.001) and *A. reconditum* (Chi-square test, χ^2^ = 7.05, *df* = 2, *P* = 0.03) infection. Sex was not a risk factor (Table 3).

**Table 3:** Number and percentage of dogs in Iran positive for *Dirofilaria immitis* and *Acanthocheilonema reconditum* DNA (*n* = 344) according to their sex, age and sampling area

Variables	No. (%)	*Dirofilaria immitis*			*Acanthocheilonema reconditum*		
		Number of infected dogs (%)	95% CI^a^	Chi-square *df*^b^ *P*-value	Number of infected dogs (%)	95% CI	Chi-square *df* *P*-value
Sex							
Male	198 (57.6)	41 (20.07)	15.3–27.02	*χ*^2^ = 0.0001 *df* = 1 *P* = 0.9	5 (2.5)	0.8–5.8	*χ*^2^ = 0.074 *df* = 1 *P* = 0.06
Female	146 (42.4)	34 (23.3)	16.7–30.9		0	0	
Age							
< 1 year	62 (18.02)	11 (17.7)	9.2–29.5	*χ*^2^ = 14.6 *df* = 2 *P* = 0.001*	0	0	*χ*^2^ = 7.05 *df* = 2 *P* = 0.03*
≥ 1 year to < 5 years	138 (40.1)	47 (34.1)	26.2–42.6		0	0	
≥ 5 years	144 (41.9)	17 (11.8)	7.0–18.2		5 (3.5)	1.1–7.9	
Geographical origin							
Esfahan	105 (30.5)	1 (0.95)	0.02–5.2	*χ*^2^ = 77.7 *df* = 4 *P* = 0.0001*	1 (0.95)	0.02–5.2	*χ*^2^ = 7.6 *df* = 4 *P* = 0.1
Lorestan	101 (29.4)	7 (6.9)	2.8–13.8		0	0	
Mazandaran	66 (19.2)	33 (50)	37.4–62.6		3 (4.5)	0.95–12.7	
Qazvin	44 (12.8)	12 (27.3)	14.9–42.8		0	0	
Gilan	28 (8.1)	22 (78.6)	59.05–91.7		1 (3.6)	0.09–18.3	
Total	344	75 (21.8)	17.5–26.5		5 (1.45)	0.47–3.36	

A *p*-value less than 0.05 is statistically significant

^a^Confidence interval

^b^Degrees of freedom

*Statistically significant

Consensus sequences for *D. immitis* and *A. reconditum* displayed 100% nucleotide identity with those available in the GenBank^®^ database. In particular, *D. immitis* nucleotides were identical to AJ537512 (dog from Australia), MK250758 (dog from Thailand) and MH920260 (human from Iran). In addition, *A. reconditum* nucleotides showed 100% similarity with isolates from dogs in Moldova (MW656249), Portugal (MW246127), Algeria (MW138007) and Italy (JF461456).

**Fig. 2:**
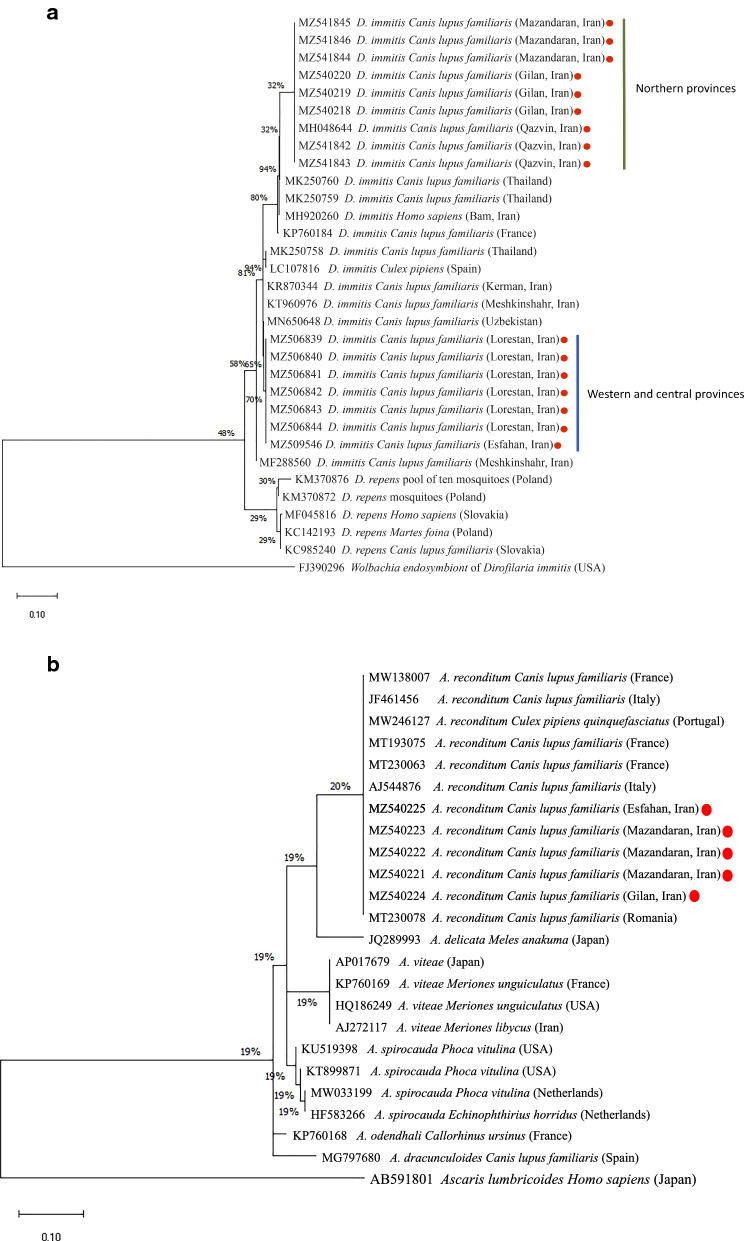
Phylogenetic relationship of *D. immitis* (**a**) and *A. reconditum* (**b**) sequences isolated in this study to other filarial helminths based on a partial sequence of the *cox*1 gene. Homologous sequences from *Wolbachia* endosymbiont of *D. immitis* and *Ascaris lumbricoides* (GenBank: FJ390296, AB591801) were used as outgroups

In the ML tree based on the partial *cox*1 gene sequences of *D. immitis*, all representative sequences of northern provinces (Mazandaran, Gilan, Qazvin) were grouped together and distinctly separate from western and central provinces (Lorestan and Esfahan) (Fig. 2a). The ML tree based on the partial *cox*1 gene sequences of *A. reconditum* was supported by the distinct separation from other filarial nematodes inferred from the phylogenetic analyses (Fig. 2b). The ML tree based on the partial *ftsZ* gene sequences of *Wolbachia* endosymbiont of *D. immitis* showed that all sequences detected herein clustered in one clade (supergroup C) and distinct from *Wolbachia* from *Brugia* spp. (supergroup D).

## Discussion

The detection of filarial parasites in the blood of dogs in different regions of Iran, including *D. immitis* with zoonotic importance, indicates that all dog populations in the country are exposed to these VBDs, thus posing a risk not only to them but also to public health. Since the distribution pattern and positivity of dogs for VBDs differ in different areas [30, 31], updated epidemiological data are necessary for the design and implementation of operational control strategies. This study fills the gap regarding microfilaraemia in dogs of two Iranian provinces, and records the highest prevalence of dirofilariosis ever reported from Iran (i.e. 22/28 dogs in Gilan province in the north). Dogs of four other provinces were also infected, albeit at a lower prevalence.

The trend of positivity for *D. immitis* in different provinces of Iran paralleled that of the climatic and ecological features, with the highest prevalence recorded in Gilan (78.6%) and Mazandaran (50%) in the Caspian region, which could be due to the typical Mediterranean climate all year-round [32]. The low prevalence observed in Esfahan (0.9%), Lorestan (6.9%) and Qazvin (27.3%) could be due to the increase in temperature and decrease in yearly rainfall.

This study provides new information about the microfilaraemia in dogs from three Iranian provinces (i.e. Esfahan, Lorestan and Qazvin) and updates the epidemiology of the infections in two provinces (i.e. Mazandaran and Gilan). In Mazandaran, we found infection with *D. immitis* in 22% and with *A. reconditum* in 4.5% of dogs by PCR. Previous studies in this province demonstrated infection with *D. immitis* in up to 15.2% of dogs based on microscopic examination of blood, and 60.9% based on necropsy [7, 33]. However, in a later study performed in 75 dogs (2018–2019) in the same province, no infected animals were diagnosed by PCR [34]. Differences in reported infection rates in different years could be due to changes in the environment, bioclimatic factors such as rainfall which affect the propagation of intermediate arthropod hosts, and differences in the population structure (e.g. age) of the examined animals [35]. On the other hand, in Gilan province we detected *D. immitis* in 78.6% and *A. reconditum* in 3.6% of the examined dogs, which is close to those previously reported (i.e. 51.5% for *D. immitis* and 4.5% for *A. reconditum*) [19].

**Fig. 3:**
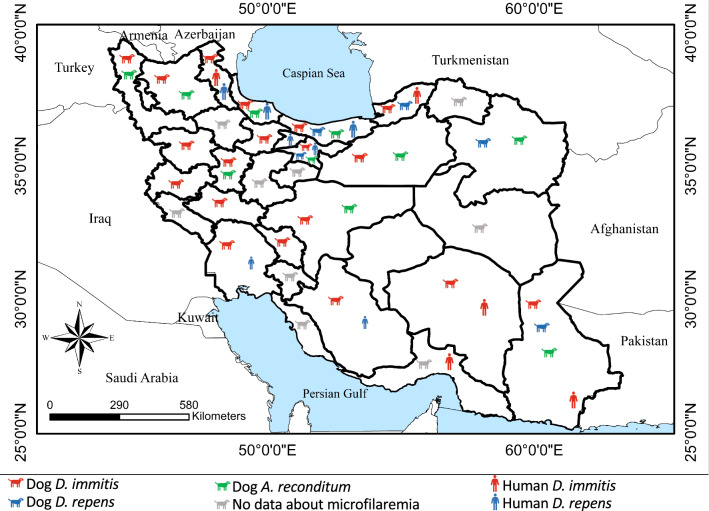
Distribution of *D. immitis*, *D. repens* and *A. reconditum* in dogs and humans of Iran. See Table 3 for data about dogs and references [8, 9, 70] for data about human cases

Since the first record of *D. immitis* infection in dogs of northern Iran [7], several studies have reported prevalence up to 62.8% for dirofilariosis (Table 4). Though there is no information about probable infection of dogs in nine out of 31 Iranian provinces with *Dirofilaria* species, reports of human cases of infection with *D. immitis* and *D. repens* in Alborz and Hormozgan indicate the occurrence of infection in the canine population as well (Fig. 3). In addition, since wild carnivores such as wolves, jackals and foxes play significant roles in the epidemiology of dirofilariosis [36, 37], more epidemiological surveillance studies are necessary for filling the gaps in knowledge about wild reservoir hosts.

**Table 4:** Prevalence of haematic microfilariae in dogs of Iran (*n* = 6520) until September 2021. Highest records are marked in bold

Region	Province	No. examined	Method	*Dirofilaria immitis* infection rate (%)	*Dirofilaria repens* infection rate (%)	*Acanthocheilonema reconditum* infection rate (%)	Year of study	References
North	Mazandaran	25	Necropsy	4	**60.9**	NS^a^	1968	[7]
		80	Microscopy	15.2	0	2.5	2004–2005	[33]
		220	Microscopy	10.9	0	0.9	2006–2008	[19]
		65	Microscopy	7.7	Microscopy: 0	Microscopy: 0	2009	[32]
			Serology	4.6				
		66	Microscopy	12.1	0	1.5	2016–2018	This study
			PCR	33	0	4.5		
		75	PCR	0	0	0	2018–2019	[34]
	Gilan	101	Microscopy	51.5	0	4.5	2006–2008	[19]
		70	Microscopy	42.8	Microscopy: 0	Microscopy: 0	2009	[32]
			Serology	51.4				
		27	Necropsy	25.9	NS	NS	2015	[38]
		28	Microscopy	39.3	0	3.6	2016–2018	This study
			PCR	**78.6**	0	3.6		
	Golestan	110	Microscopy	15.4	4.5	0	2006–2008	[19]
		65	Microscopy	10.8	Microscopy: 0	Microscopy: 0	2009	[32]
			Serology	13.8				
Northwest	West Azerbaijan	357	Microscopy	8.7	0	5.04	2001	[39]
		20	Necropsy	10	NS	NS	2010	[40]
		160	Microscopy	25.0	0	0	2010	[41]
		100	Microscopy	3	Microscopy: 0	Microscopy: 0	2014	[42]
			Serology	0				
	East Azerbaijan	80	Microscopy	25	0	0	NS	[43]
		100	Microscopy	30	0	0	2005–2006	[44]
		384	Serology	13.5	NS	NS	2011	[45]
		205	Microscopy	Not clear	0	Not clear	2010–2011	[46]
			PCR	26.2		**19.5**		
		121	Microscopy	0	Microscopy: 0	Microscopy: 0	2011–2012	[47]
			PCR	11.6				
		100	Microscopy	14	0	0	NS	[48]
		200	Microscopy	15	0	0	2017	[49]
	Ardebil	30	Necropsy	26.7	NS	NS	1987	[50]
		286	Microscopy	34.6	NS	NS	1992	[51]
		91	Microscopy	20.9	0	0	2009–2011	[52]
		15	Necropsy	13.3				
		43	Serology	62.8	NS	NS	2017	[15]
West	Kordestan	74	Microscopy	8.1	0	0	2004–2005	[53]
	Kermanshah	120	Microscopy	18.3	0	0	2011	[10]
		51	PCR	0	0	0	2018–2019	[34]
	Hamedan	157	Microscopy	4.4	Microscopy: 0	9.5	2012	[21]
			PCR	4.4		9.5		
		81	PCR	0	0	0	2018–2019	[34]
	Lorestan	101	Microscopy	0	0	0	2016–2018	This study
			PCR	6.9	0	0		
	Chaharmahal-va-Bakhtiari	69	Necropsy	1.5	NS	NS	2007–2009	[54]
Southwest	Khuzestan	23	Necropsy	8.7	NS	NS	1997	[55]
		119	Microscopy	12.6	0	0	NS	[56]
		100	Microscopy	5	Microscopy: 0	Microscopy: 0	2007–2008	[57]
			Serology	6				
		200	Microscopy	8	Microscopy: 0	Microscopy: 0	2011–2012	[58]
			Serology	9.5				
		69	PCR	0	0	0	2018–2019	[34]
East	Khorasan Razavi	138	Microscopy	0	6.4	5	1996–1997	[17]
Southeast	Kerman	100	Serology	10	Microscopy: 0	Microscopy: 0	2008	[59]
			Microscopy	2				
		98	Necropsy	0				
		33	Microscopy	Not clear	Microscopy: 0	Microscopy: 0	2013	[60]
			Serology	15.1				
		149	Microscopy	2.7	Microscopy: 0	Microscopy: 0	2013	[61]
			Serology	5.4				
			PCR	4.02				
		100	Microscopy	4	Microscopy: 0	Microscopy: 0	2017–2018	[62]
			Serology	10				
	Sistan-va-Baluchestan	87	Microscopy	Not clear	Microscopy: 0	Microscopy: 0	2013	[60]
			Serology	27.6				
		60	Microscopy	8.3	0	1.7	2013	[63]
		99	Microscopy	30.3	0	0	2017	[64]
Centre	Tehran	139	Microscopy	0	0	4.3	NS	[18]
		138	Microscopy	1.42	0	8.7	1998–1999	[65]
		311	Microscopy	0	0	0	2017	[16]
			PCR	2.3	26	NS		
	Qazvin	44	Microscopy	2.3	0	0	2016–2018	This study
			PCR	27.3	0	0		
	Esfahan	105	Microscopy	0.9	0	0.9	2016–2018	This study
			PCR	0.9	0	0.9		
	Semnan	122	Microscopy	13.1	0	2.4	NS	[66]
		112	Microscopy	5.3	0	0	2014	[67]
	Yazd	78	PCR	0	0	0	2018–2019	[34]
South	Fars	114	Microscopy	9.6	0	0	1995	[68]
		105	Necropsy	0.9	NS	NS	1998–1999	[69]

^a^NS: not stated

In this study, *A. reconditum* was detected in three out of five studied provinces, with only 1–3 infected dogs in each province. Microfilariae of *A. reconditum* were identified for the first time in the blood of 6/139 tested dogs in Tehran [18]. Since then, different studies have reported infection with *A. reconditum* in up to 19.5% of dogs [46] (Table 4; Fig. 3). Since wild canine species, such as jackals, may contribute to the epidemiology of *A. reconditum* [71], it is suggested that this less pathogenic nematode should be screened in wildlife parasitological studies.

PCR was found to detect more infected dogs in comparison with modified Knott’s test (i.e. 75 vs 21 for *D. immitis* and 5 vs 3 for *A. reconditum*), which could be due to low parasitaemia [72, 73]. The sensitivity of the two PCR methods for the detection of *D. immitis* by the *cox*1 gene and *Wolbachia* endosymbionts *ftsZ* was identical. Although the modified Knott’s test which is based on the detection and identification of microfilariae is the most popular parasitological method among concentration tests, serological assays for the detection of antibodies against *D. immitis* (e.g. enzyme-linked immunosorbent assay [ELISA] and immunochromatographic tests) are also common [72, 74]. Antigen tests may detect occult infections such as amicrofilaraemic cases [72], but positive dogs could have overcome the infection or were only exposed to *D. immitis* in the past without the development of actual infection [75].

Phylogenetic analysis of *D. immitis* nucleotide sequences in this study showed that Iranian isolates from dogs living in northern provinces (i.e. Mazandaran, Gilan and Qazvin) clustered together, and isolates from dogs of Lorestan and Esfahan clustered separately. This could be due to differences in the population of the parasites, segregated in specific geographical areas or in dog populations. However, due to the relatively low number of animals, any hypothesis would need confirmatory evidence. It has been suggested that relocation of domestic dogs within and between countries is associated with the risk of dissemination of parasites and vectors, and hence continuous relocation represents a global veterinary and public health concern [76], mainly in low- or middle-income countries where the public health politics are disregarded [77, 78]. In this context, and considering that canine dirofilariosis is significantly more prevalent in the Caspian region, veterinary examination of dogs prior to movement to other regions of the country is advocated.

## Conclusions

The present study confirmed HWD in three more Iranian provinces (i.e. Lorestan, Qazvin and Esfahan). A review of the previous reports shows that dogs in all areas of Iran are at risk of dirofilariosis. Considering that *D. immitis* is a zoonotic parasite, the application of ectoparasiticides and repellents is highly recommended for dogs in Iran. Furthermore, a national program for the control of VBDs in stray dogs in Iran is necessary.

## Data Availability

All data generated or analysed during this study are included in this published article. Representative sequences of pathogens detected in this study were deposited in the GenBank^®^ database under the accession numbers MZ541842–MZ541846, MZ540218–MZ540220, MZ506839–MZ506844, MH048644, MZ509546 for *D. immitis*, MZ540221–MZ540225 for *A*. *reconditum*, and MZ566793–MZ566801 for *Wolbachia* endosymbiont of *D. immitis*.

## Abbreviations

*cox*1: Cytochrome *c* oxidase subunit 1
*ftsZ*: Filamenting temperature-sensitive mutant Z
HWD: Heartworm disease
PCR: Polymerase chain reaction
OR: Odds ratio
DNA: Deoxyribonucleic acid
CI: Confidence interval
ELISA: Enzyme-linked immunosorbent assay
*df*: Degrees of freedom

## References

[1] Dantas-Torres F, Otranto D (2020). Overview on *Dirofilaria immitis* in the Americas, with notes on other filarial worms infecting dogs. Vet Parasitol.

[2] Dantas-Torres F, Otranto D (2013). Dirofilariosis in the Americas: a more virulent *Dirofilaria immitis*?. Parasit Vectors.

[3] Baneth G, Thamsborg S, Otranto D, Guillot J, Blaga R, Deplazes P (2016). Major parasitic zoonoses associated with dogs and cats in Europe. J Comp Pathol.

[4] Brianti E, Gaglio G, Napoli E, Giannetto S, Dantas-Torres F, Bain O (2012). New insights into the ecology and biology of *Acanthocheilonema reconditum* (Grassi, 1889) causing canine subcutaneous filariosis. Parasitol.

[5] Napoli E, Brianti E, Falsone L, Gaglio G, Foit S, Abramo F (2014). Development of *Acanthocheilonema reconditum* (Spirurida, Onchocercidae) in the cat flea *Ctenocephalides felis* (Siphonaptera, Pulicidae). Parasitol.

[6] Huynh T, Thean J, Maini R (2001). *Dipetalonema reconditum* in the human eye. Br J Ophthalmol.

[7] Sadighian A (1969). Helminth parasites of stray dogs and jackals in Shahsavar area, Caspian region. Iran J Parasitol.

[8] Azari-Hamidian S, Yaghoobi-Ershadi M, Javadian E, Moubedi I, Abai M (2007). Review of dirofilariasis in Iran. J Guilan Univ Med Sci..

[9] Azari-Hamidian S, Norouzi B, Harbach RE (2019). A detailed review of the mosquitoes (Diptera: Culicidae) of Iran and their medical and veterinary importance. Acta Trop.

[10] Bohloli Oskoii S, Sadeghi E, Hashemian AH, Ghaffari KS (2013). Study on shepherd dog dirofilariosis in Kermanshah province in 2011–2012. J Vet Lab Res.

[11] Parsa R, Sedighi A, Sharifi I, Bamorovat M, Nasibi S (2020). Molecular characterization of ocular dirofilariasis: a case report of *Dirofilaria immitis* in south-eastern Iran. BMC Infect Dis.

[12] Azari-Hamidian S, Yaghoobi-Ershadi M, Javadian E, Abai M, Mobedi I, Linton YM (2009). Distribution and ecology of mosquitoes in a focus of dirofilariasis in northwestern Iran, with the first finding of filarial larvae in naturally infected local mosquitoes. Med Vet Entomol.

[13] Khanzadeh F, Khaghaninia S, Maleki-Ravasan N, Koosha M, Oshaghi MA (2020). Molecular detection of *Dirofilaria* spp. and host blood-meal identification in the *Simulium turgaicum* complex (Diptera: Simuliidae) in the Aras River Basin, northwestern Iran. Parasit Vectors.

[14] Anvari D, Narouei E, Daryani A, Sarvi S, Moosazadeh M, Hezarjaribi HZ (2020). The global status of *Dirofilaria immitis* in dogs: a systematic review and meta-analysis based on published articles. Res Vet Sci.

[15] Khanmohammadi M, Falak R, Meamar AR, Arshadi M, Akhlaghi L, Razmjou E (2019). Molecular detection and phylogenetic analysis of endosymbiont *Wolbachia pipientis* (Rickettsiales: Anaplasmataceae) isolated from *Dirofilaria immitis* in northwest of Iran. J Arthropod Borne Dis.

[16] Pedram N, Shojaee Tabrizi A, Hosseinzadeh S, Pourmontaseri M, Rakhshandehroo E (2019). Prevalence of *Dirofilaria immitis* and *Dirofilaria repens* in outdoor dogs in Tehran Province, Iran. Comp Clin Path.

[17] Razmi G (1999). A study of the situation of different filarial infection in dogs of Mashhad. J Fac Vet Med Univ Tehran..

[18] Niak A, Khatibi S (1971). A record of *Dipetalonema* (*Acanthocheilonema*) (Filariata: Setariidae) in dogs in Iran. Vet Rec.

[19] Ranjbar-Bahadori S, Veshgini A, Shirani D, Eslami A, Mohieddin H, Shemshadi B (2011). Epidemiological aspects of canine dirofilariasis in the north of Iran. Iran J Parasitol.

[20] Ranjbar-Bahadori S, Eslami A, Bokaic S (2007). Evaluation of different methods for diagnosis of *Dirofilaria immitis*. Pak J Biol Sci.

[21] Hoseini M, Jalousian F, Hoseini SH, Sadeghian AG (2020). A cross sectional study on *Dirofilaria immitis* and *Acanthocheilonema reconditum* in sheepdogs in a western region in Iran. Vet Res Forum.

[22] Stratton SJ (2021). Population research: convenience sampling strategies. Prehosp Disaster Med.

[23] Eslami A, Meshgi B (2000). Periodicity of *Dirofilaria immitis* in a dog from Tehran. J Fac Vet Med Univ Tehran..

[24] Ionică AM, Matei IA, D’Amico G, Bel LV, Dumitrache MO, Modrý D (2017). *Dirofilaria immitis* and *D. repens* show circadian co-periodicity in naturally co-infected dogs. Parasit Vectors.

[25] Rojas A, Rojas D, Montenegro VM, Baneth G (2015). Detection of *Dirofilaria immitis* and other arthropod-borne filarioids by an HRM real-time qPCR, blood-concentrating techniques and a serological assay in dogs from Costa Rica. Parasit Vectors.

[26] Albonico F, Loiacono M, Gioia G, Genchi C, Genchi M, Mortarino M (2014). Rapid differentiation of *Dirofilaria immitis* and *Dirofilaria repens* in canine peripheral blood by real-time PCR coupled to high resolution melting analysis. Vet Parasitol.

[27] Turba ME, Zambon E, Zannoni A, Russo S, Gentilini F (2012). Detection of *Wolbachia* DNA in blood for diagnosing filaria-associated syndromes in cats. J Clin Microbiol.

[28] Kumar S, Stecher G, Li M, Knyaz C, Tamura K (2018). MEGA X: molecular evolutionary genetics analysis across computing platforms. Mol Biol Evol.

[29] Tamura K, Nei M (1993). Estimation of the number of nucleotide substitutions in the control region of mitochondrial DNA in humans and chimpanzees. Mol Biol Evol.

[30] Mendoza-Roldan J, Benelli G, Panarese R, Iatta R, Furlanello T, Beugnet F (2020). *Leishmania infantum* and *Dirofilaria immitis* infections in Italy, 2009–2019: changing distribution patterns. Parasit Vectors.

[31] Brianti E, Panarese R, Napoli E, De Benedetto G, Gaglio G, Bezerra-Santos MA (2021). *Dirofilaria immitis* infection in the Pelagie archipelago: the southernmost hyperendemic focus in Europe. Transbound Emerg Dis.

[32] Malmasi A, Hosseini S, Aramoon M, Bahonar A, Seifi HA (2011). Survey of canine *Dirofilaria immitis* infection in Caspian provinces of Iran. Iran J Vet Res.

[33] Bahadori SR, Mohtasham RM, Eslami A, Meshgi B (2005). Study on blood filariosis of dog in Tonekabon. J Fac Vet Med Univ Tehran.

[34] Iatta R, Sazmand A, Nguyen V-L, Nemati F, Bahiraei Z, Mazhar Ayaz M (2021). Vector-borne pathogens in dogs of different regions of Iran and Pakistan. Parasitol Res.

[35] Alho AM, Landum M, Ferreira C, Meireles J, Goncalves L, de Carvalho LM (2014). Prevalence and seasonal variations of canine dirofilariosis in Portugal. Vet Parasitol.

[36] Otranto D, Deplazes P (2019). Zoonotic nematodes of wild carnivores. Int J Parasitol Parasites Wildl.

[37] Meshgi B, Eslami A, Bahonar A, Kharrazian-Moghadam M, Gerami-Sadeghian A (2009). Prevalence of parasitic infections in the red fox (*Vulpes vulpes*) and golden jackal (*Canis aureus*) in Iran. Iran J Vet Res.

[38] Vafae Eslahi A, Kia EB, Mobedi I, Sharifdini M, Badri M, Mowlavi G (2017). Road killed carnivores illustrate the status of zoonotic helminthes in Caspian Sea littoral of Iran. Iran J Parasitol.

[39] Meshgi B, Eslami A, Ashrafi HJ (2002). Epidemiological survey of blood filariae in rural and urban dogs of Tabriz. J Fac Vet Med Univ Tehran..

[40] Khezri A, Tavasoli M, Javadi S, Bartafteh E. Prevalence of parasites of the cardiovascular system and genitourinary system in stray dogs in Urmia. In: 16th Iranian Veterinary Congress. Tehran, Iran. 2010: THVC16_0802.

[41] Javadi S, Hanifeh M, Tavassoli M, Dalir Naghadeh B, Khezri A, Hadian M (2011). Dirrofilariasis in shepherd dogs of high altitudes areas in West Azerbaijan-Iran. Vet Res Forum.

[42] Naghipour M, Javadi S, Tavassoli M, Shamsi S (2018). Serological study of *Dirofilaria immitis* in urban dogs of Urmia using modified Knott and rapid antigen tests. J Zoon Dis.

[43] Amouoghli Tabrizi B, Dastmalchi F, Gharadagy Y, Khakpour M (2007). Evaluation of some hematological changes in canine heartworm infection (*Dirofilaria immitis*). J Vet Clin Pathol.

[44] Nematollahi A, Javidi Barazandeh MA (2010). A survey on *Dirofilaria immitis* occurence in stray dogs of Tabriz (Iran). Acta Vet Brno.

[45] Khanmohammadi M (2012). *Dirofilaria immitis* occult infection and seropervalence in sheepdogs in Sarab (East Azerbaijan Province). J Comp Pathol.

[46] Razmaraii N, Sadegh-Eteghad S, Babaei H, Paykari H, Esmaeilnia K, Froghy L (2013). Molecular survey of canine microfilariae species in East-Azerbaijan province of Iran. Arch Razi Inst.

[47] Hosseinzadeh Varjoy M, Ashrafi Helan J, Salehi N, Bazmani A, Nematollahi A, Imani BA (2016). Molecular detection and epidemiological aspects of *Dirofilaria immitis* in dogs in Tabriz and suburbs. J Mazandaran Univ Med Sci.

[48] Raoof P, Garedaghi Y (2017). Investigation of infection with *Dirofilaria immitis* parasite in stray dogs in Tabriz city of Iran. Livest Sci.

[49] Hashemzadeh Farhang H, Bahavarnia SR, Esmailzadeh MJ, Mahmoudi KN (2020). Survey on zoonotic importance and prevalence of *Dirofilaria immitis* Infection in dogs of Tabriz. Iran Int J Med Parasitol Epidemiol.

[50] Mobedi I, Javadian E, Abaei M. Introducing the foci of zoonotic dog heartworm (*Dirofilaria immitis*) (Nematoda, Filaioidea) in Meshginshahr region (East Azerbaijan Province) and its importance in Iran. In: *First National Iranian Congress of Parasitology*. Rasht, Iran. 1990:78.

[51] Bokaei S, Mobedi I, Mohebali M, Hosseini SH, Nadim A (1998). A study of dirofilarisis prevalence in dogs in Meshkinshahr area, Northwest Iran. J Fac Vet Med Univ Tehran..

[52] Zarei Z, Kia EB, Heidari Z, Mikaeili F, Mohebali M, Sharifdini M (2016). Age and sex distribution of *Dirofilaria immitis* among dogs in Meshkin-Shahr, northwest Iran and molecular analysis of the isolates based on COX1 gene. Vet Res Forum.

[53] Yakhchali M, Sadi F (2008). Prevalence of *Dirofilaria immitis* infections in dogs in Saghez city, Kurdistan province, Iran. Vet Res Biol Prod.

[54] Namjoo A, Vahed-Dehkordi E, Jafarian-Dehkorid M. Prevalence of *dirofilaria* infection in necropsied dogs in Shahrekord. In: First National Congress of Veterinary Laboratory Sciences. Tehran, Iran 2009: VETLAB01_455.

[55] Farahnak A, Mobedi I, Mohamadi F (1998). Study of zoonotic helminths of carnivores in Khuzestan, Iran. Iran J Public Health.

[56] Ranjbar-Bahadori S, Delvarzadeh E, Shemshadi B (2009). *Dirofilaria immitis* infection in stray dogs of Khuzestan, a province in South-Western Iran. Iran J Vet Med.

[57] Razi Jalali MH, Alborzi A, Avizeh R, Mosallanejad B (2010). A study on *Dirofilaria immitis* in healthy urban dogs from Ahvaz, Iran. Iran J Vet Res.

[58] Razi Jalali M, Ghorbanpoor M, Mosallanejad B, Avizeh R, Alborzi A, Rahrowani M (2013). Diagnosis of *Dirofilaria immitis* infection in urban and rural dogs in Ahvaz city by counterimmunoelectrophoresis. J Fac Vet Med Univ Tehran..

[59] Akhtardanesh B, Radfar MH, Voosough D, Darijani N (2011). Seroprevalence of canine heartworm disease in Kerman, southeastern Iran. Comp Clin Path.

[60] Khedri J, Radfar MH, Borji H, Azizzadeh M, Akhtardanesh B (2014). Canine heartworm in southeastern of Iran with review of disease distribution. Iran J Parasitol.

[61] Bamorovat M, Sharifi I, Fasihi-Harandi M, Nasibi S, Sadeghi B, Khedri J (2017). Parasitological, serological and molecular study of *Dirofilaria immitis* in domestic dogs, Southeastern Iran. Iran J Parasitol.

[62] Naderi A, Sharifi I, Aflatoonian MR, Mostafavi M, Parizi MH, Mashayekhi J (2021). Dirofilariosis caused by Dirofilaria immitis in the south of Kerman province, Iran. Microb Pathog.

[63] Mohebby M (2016). Investigate the prevalence of *Dirofilaria immitis* in dogs in the city of Zabol.

[64] Anvari D, Saadati D, Siyadatpanah A, Gholami S (2019). Prevalence of dirofilariasis in shepherd and stray dogs in Iranshahr, southeast of Iran. J Parasit Dis.

[65] Meshgi B, Eslami A (2001). Investigation on the filariosis of dog in Tehran area. J Fac Vet Med Univ Tehran..

[66] Ranjbar-Bahadori S, Hekmatkhah A (2007). A study on filariosis of stray dogs in Garmsar. J Fac Vet Med Univ Tehran..

[67] Salimi Bejestani M, Darabizade Z (2019). Determining the prevalence of blood’s microfilaria in dogs of Semnan city by modified Knott test. J Vet Lab Res..

[68] Jafari S, Gaur S, Khaksar Z (1996). Prevalence of *Dirofilaria immitis* in dogs of Fars province of Iran. J Appl Anim Res.

[69] Sadjjadi S, Mehrabani D, Oryan A (2004). Dirofilariosis of stray dogs in Shiraz, Iran. J Vet Parasitol.

[70] Ghasemi E, Shamsinia S, Taghipour A, Anvari D, Bahadory S, Shariatzadeh SA (2020). Filarial worms: a systematic review and meta-analysis of diversity in animals from Iran with emphasis on human cases. Parasitology.

[71] Gherman CM, Mihalca AD (2017). A synoptic overview of golden jackal parasites reveals high diversity of species. Parasit Vectors.

[72] Panarese R, Iatta R, Mendoza-Roldan JA, Szlosek D, Braff J, Liu J (2020). Comparison of diagnostic tools for the detection of *Dirofilaria immitis* infection in dogs. Pathogens.

[73] Mendoza-Roldan JA, Gabrielli S, Cascio A, Manoj RR, Bezerra-Santos MA, Benelli G (2021). Zoonotic *Dirofilaria immitis* and *Dirofilaria repens* infection in humans and an integrative approach to the diagnosis. Acta Trop.

[74] Solgi R, Sadjjadi SM, Mohebali M, Zarei Z, Golkar M, Raz A (2018). Development of new recombinant DgK antigen for diagnosis of *Dirofilaria immitis* infections in dogs using ELISA technique and its comparison to molecular methods. Iran Biomed J.

[75] Selim A, Alanazi AD, Sazmand A, Otranto D (2021). Seroprevalence and associated risk factors for vector-borne pathogens in dogs from Egypt. Parasit Vectors.

[76] Wright I, Jongejan F, Marcondes M, Peregrine A, Baneth G, Bourdeau P (2020). Parasites and vector-borne diseases disseminated by rehomed dogs. Parasit Vectors.

[77] Otranto D, Dantas-Torres F, Mihalca AD, Traub RJ, Lappin M, Baneth G (2017). Zoonotic parasites of sheltered and stray dogs in the era of the global economic and political crisis. Trends Parasitol.

[78] Dantas-Torres F, Otranto D (2016). Best practices for preventing vector-borne diseases in dogs and humans. Trends Parasitol.

